# Children’s outcomes in road traffic accidents: challenges for personal injury assessment

**DOI:** 10.1093/fsr/owad034

**Published:** 2023-09-22

**Authors:** Flávia Cunha-Diniz, Tiago Taveira-Gomes, José M Teixeira, Teresa Magalhães

**Affiliations:** Legal Medicine and Forensic Sciences Unit, Department of Public Health and Forensic Sciences and Medical Education, Faculty of Medicine of the University of Porto, Porto, Portugal; Centre for Health Technology and Services Research (CINTESIS), Faculty of Medicine, University of Porto, Porto, Portugal; Instituto Universitário de Ciências da Saúde – CESPU (IUCS – CESPU), Gandra, Portugal; MTG Research and Development Lab, Porto, Portugal; Faculty of Health Sciences, University Fernando Pessoa (FCS-UFP), Porto, Portugal; Porto Health Care Unity - Accidents, Fidelidade - Insurance Company, Porto, Portugal; Centre for Health Technology and Services Research (CINTESIS), Faculty of Medicine, University of Porto, Porto, Portugal; Instituto Universitário de Ciências da Saúde – CESPU (IUCS – CESPU), Gandra, Portugal; MTG Research and Development Lab, Porto, Portugal; Porto Health Care Unity - Accidents, Fidelidade - Insurance Company, Porto, Portugal

**Keywords:** road traffic accident, medico-legal evaluation, injury assessment, damage assessment, outcomes, children

## Abstract

Children represent a specific group of road traffic accident (RTA) victims. Performing a personal injury assessment (PIA) on a child presents a significant challenge, especially when assessing permanent disabilities and needs. However, medico-legal recommendations for PIA in such cases are lacking. The main objective of this study was to analyse the differences between children and a young- and middle-aged adult population of RTA victims to contribute to the development of relevant guidelines. Secondary objectives were to identify and characterize specifics of children’s posttraumatic damages regarding: (i) temporary and permanent outcomes; and (ii) medico-legal damage parameters in the Portuguese context. We performed a retrospective study of RTA victims by comparing two groups (*n* = 114 each) matched for acute injury severity (SD = 0.01): G1 (children) and G2 (young- and middle-aged adults). Logistic regression was used to estimate the odds ratios. G1 presented a greater chance of evolving without or with less severe *body*, *functional* and *situational* outcomes (three-dimensional assessment methodology), and with lower permanent functional disability values than G2. Our findings suggest that childhood trauma generally has a better prognosis than trauma in young- and middle-aged adults. This study generated evidence on the subject and highlighted the most significant difficulties encountered by medico-legal experts when performing PIA in children.

**Key points:**

- This retrospective study of PIA in child victims of RTA in Portugal considered outcomes in victims’ real-life situations.
- Several significant differences between children and young- and middle-aged adults were observed.
- Children's cases presented better results in terms of the severity of *body*, *functional* and *situational* outcomes, and permanent damage parameters.
- The average time between the RTA and final PIA date and the consolidation time were longer for children because of the need to wait for the Children’s next growth phase or final pubertal period (as applicable), which increased the time for PIA conclusion.
- There were several difficulties in the medical-legal evaluation of children’s cases, which was a complex process because the trauma affected them in their growth phase.

## Introduction

Accidental injury is a leading cause of death and acquired disability in children [1–7]. In the USA, the overall paediatric trauma survival rate ranges from 80% to 95% [1–4, 8]. Statistics from the Netherlands show that for every child killed in a road traffic accident (RTA), another 42 are seriously injured [9]. In Portugal, among the 5 700 children who were RTA victims in 2019, 0.6% died, 3.5% were seriously injured, and 95.9% suffered minor injuries [10].

Nonfatal injuries, even those that are minor, can have significant short- and long-term corporal and psychological outcomes that are associated with significant losses for the individual in all life contexts, including quality of life. These injuries represent a major cause of temporary and permanent disability and have a significant negative impact on families and community networks [1–4, 6, 8, 11–13]. Children are an especially vulnerable group in the context of road traffic. In particular, younger children have an increased risk for being run over by a vehicle because despite having the necessary motor skills to walk on the streets, they lack cognitive, sensory, and behavioural perception skills to perceive traffic and associated risks and understand the meaning of road signs [14, 15]. Being run over by a vehicle is the leading cause of death and disability for children in multiple countries [16, 17], and the resulting injuries tend to be worse than those suffered when inside motor vehicles [18]. Furthermore, children suffer different injuries than adults because: (i) they have less mass than adults and their kinetic force is reduced, causing a lower-intensity accident; and (ii) children tend to sit in the rear seats of vehicle, where they are more protected. Therefore, injury patterns differ between children and adults. Children tend to have fewer thoracic, intraabdominal, pelvic, and long bone injuries and have a lower Injury Severity Score (ISS) despite a higher Glasgow Coma Scale score [15, 19]. In addition, the evolution and severity of injuries and their outcomes tend to have a better prognosis in children than in adults [20, 21]. These results may be explained by a better adaptive process and greater physiological plasticity with a better response to trauma, which allows children to evolve further without permanent sequelae [21]. However, when cases evolve with sequelae, it is challenging to perform personal injury assessment (PIA) for children; i.e. clarifying the concerns they have in terms of permanent disabilities and needs, which are difficult to predict for their future life.

**Table 1:** Matched sample characterization regarding ISS.

Parameter	Total (*N* = 228)	G1 (*n* = 114)	G2 (*n* = 114)	SD
$\overline{\mathrm{X}}$	9.3 ± 9.5	9.3 ± 9.4	9.3 ± 9.7	−0.001
*n* (%)				
0 (no acute lesion)	3 (1.3)	2 (1.8)	1 (0.9)	−0.006
1–8 (mild/moderate)	106 (46.5)	52 (45.6)	54 (47.4)	0.020
9–15 (serious)	77 (33.8)	39 (34.2)	38 (33.3)	−0.008
16–24 (severe)	22 (9.6)	11 (9.6)	11 (9.6)	0
≥25 (critical)	20 (8.8)	10 (8.8)	10 (8.8)	0

ISS: Injury Severity Score.

To our knowledge, there are no published medico-legal recommendations for PIA in children’s cases. Therefore, it is urgent to develop medico-legal research on these cases to support medical experts with scientific evidence. The main objective of this study was to analyse the differences between children and a young- and middle-aged adult population of RTA victims to contribute to development of guidelines on the subject. Secondary objectives were to identify and characterize specifics of children’s posttraumatic damage regarding: (i) temporary and permanent outcomes; and (ii) medico-legal damage parameters in the Portuguese context.

## Materials and methods

### Data collection methodology

This retrospective study used a convenience sample based on medico-legal reports of PIA cases from the Centro Hospitalar de Sao Joao/Faculdade de Medicina do Porto with permission for using in this study. The inclusion criteria for reports were: (i) final medico-legal reports about RTA victims showing an established causality link between the trauma and injuries; (ii) victims aged <65 years; (iii) performed at a healthcare unit of a Portuguese insurance company; (iv) performed between 2018 and 2020; and (v) assessed by three selected physicians with specialization in forensic medicine and extensive experience in PIA to ensure data reliability. All physicians were aligned with the official Portuguese rules, including the *three-dimensional methodology* for describing permanent outcomes [22–24] and the different parameters of damage in civil law for outcome quantification [22, 23]. We did not consider the victim’s sex, accident type, or type of insurance responsibility (i.e. with or without fault) at this stage.

One child case was excluded because it deviated too much from the median. This was a case in which the outcome was a persistent vegetative coma, which exaggeratedly increased the results of the mean values for children. However, this case is presented and discussed later. A database was created for this study and completed by one of the physicians who performed medico-legal assessments for cases. No information was included that could allow those involved to be identified.

Two age groups were considered: (i) G1 comprised children aged <18 years because the World Health Organization defines a child as someone under 18 years of age (unless national law defines otherwise) [25] (*n =* 114; 50%); and (ii) G2 comprised young- and middle-aged adults aged 18–64 years (*n =* 114; 50%). G2 were identified from an original sample of 431 people using propensity score matching with SPSS software (Tulsa, OK, USA). G1 corresponded to the analysed sample, and G2 corresponded to the control sample. G1 included 65 (57%) males, and the age average was 11.4 ± 4.8 years (<1 year: 1.8%; 1–4 years: 7.8%; 5–10 years: 30.8%; and ≥11 years: 59.6%).

**Figure 1:**
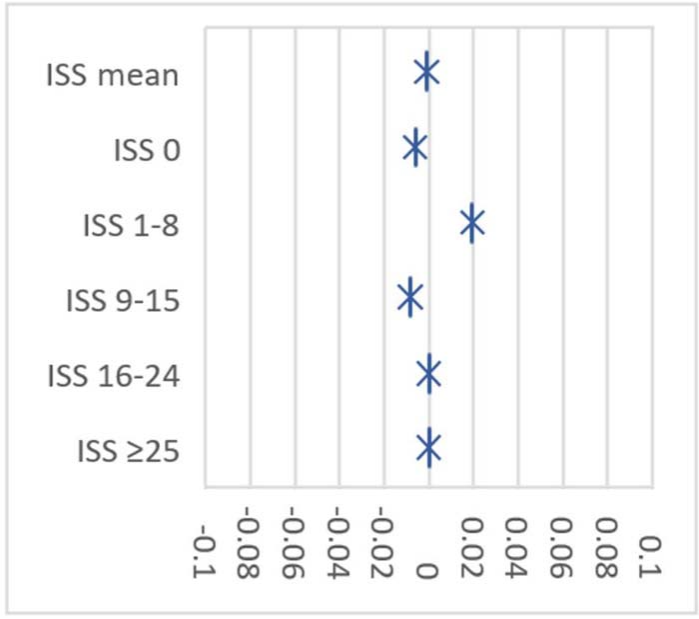
Standardized differences for matched predictors (propensity score matching).

ISS [26, 27] was used as a predictor to ensure that G1 and G2 presented a similar initial picture after the RTA. ISS was retrospectively estimated in the acute phase of each case with consideration of the clinical records. The ISS variables were categorized into five classes as shown in Table 1. To determine whether the matched samples were comparable, we used the standardized difference, which is considered balanced at ≤0.1 [28]. Therefore, as shown in Table 1 and Figure 1, our samples were balanced across all selected predictors.

### Assessment methodology

Clinical records were analysed to retrospectively estimate injury severity in the acute phase using the ISS [26, 27]. ISS variables were categorized into five classes: 0 (nonexistent), 1–8 (minor or moderate), 9–15 (serious), 16–24 (severe), and 25–75 (critical).

The *three-dimensional methodology* (*body*, *functional* and *situational* levels) was used to describe permanent outcomes based on official Portuguese rules [22–24]. This methodology includes the *Inventory for Handicap Assessment* [29], which was used to quantify permanent outcome severity. This tool has been validated for Portuguese RTA victims aged 16–65 years. We chose this tool because it is a medico-legal inventory intended for PIA purposes and to our knowledge, no other instrument has been validated for the child population to date. This tool allowed us to quantify the severity degree of *body*, *functional* and *situational* levels, and the *damage coefficient* [20, 24, 29]. This *coefficient* corresponds to the average of the final scores for each scale of the three referred levels and considers five severity groups by increasing severity. The meaning of each level is [20, 24, 29]: (i) *body* level, which assesses biological outcomes that may include morphological, anatomical, histological, physiological, and genetic particularities; (ii) *capacity/functional* level, which assesses physical and mental capacities (current or potential), taking into account age and sex irrespective of the live setting; and (iii) *life situations/participation/activities* level, which assesses the confrontation (concrete or potential) between those affected and the reality of their physical, familial, social, cultural, educational, and professional environment.

To quantify the different parameters of damage in civil law, we used Portuguese medico-legal damage parameters [22, 23] as follows.

(i) *Total temporary functional deficit*: period (days) in which the victim is prevented from autonomously performing acts of daily, family, and social life (without reference to professional activities). This parameter mostly corresponds with hospitalization time.
(ii) *Partial temporary functional deficit*: period (days) in which the victim may resume daily, family, and social life activities with some degree of autonomy, although still with limitations.
(iii) *Quantum doloris*: physical and psychic suffering experienced by the victim during the period of temporary damage based on a 7-point scale of increasing severity.
(iv) *Permanent functional deficit* (PFD): definitive effects on the victim’s physical or psychic integrity with repercussions for daily life activities, including family and social life, leisure and sporting activities, although it is independent of professional activities. This parameter is assessed on a 100-point scale of increasing severity and was categorized as 0, 1–9, 10–19, 20–39, and 40–100 based on the case distribution and severity groups.
(v) *Future damage*: damage that is not yet observable in the PIA but is certain to develop, corresponding to future aggravation of the sequelae and consequent aggravation of specific damage parameters (i.e. PFD).
(vi) *Permanent aesthetic damage*: repercussions of the sequelae on the victim’s self-image and image from others. This parameter is rated on a 7-point scale of increasing severity.
(vii) *Permanent repercussion on sporting and leisure activities*: the impossibility of the victim engaging in certain leisure, physical, or social activities that they regularly participated in and that represented a clear source of personal fulfilment and gratification. This parameter is rated on a 7-point scale of increasing severity.
(viii) *Permanent needs*: the victim’s needs, with repercussions on their independence and autonomy. This parameter should be assessed with consideration of the victim’s best chances of rehabilitation and reintegration.

We did not analyse *permanent repercussions on sexual activity* despite these being critical functions to assess. In addition, we did not analyse *temporary professional repercussions* and *permanent professional repercussions* because children can only start working from age 16 years (Decree-Law 7/2009, 12 February) in Portugal. Children in Portugal generally do not work before age 18 years (*n =* 4 in our sample).

### Data analysis

All analyses were performed using SPSS for Windows version 27.0 (IBM Corp., Armonk, NY, USA). Descriptive statistics were used to describe the study population (total and stratified by age). Chi-square tests were used to assess the dependence between the frequency variables. Continuous variables were assumed to be normally distributed. Logistic regression was used to estimate the odds ratio (OR) and 95% confidence interval (CI) for all measures of effect analysed. The OR was considered statistically significant if the CI did not exceed the value 1. In all analyses, the level of statistical significance was set at *P* < 0.05.

## Results

The average timespan between the RTA date and final PIA date was 510.1 ± 764.0 (min = 28; max = 4 476) days, and the average consolidation time was 321.8 ± 572.0 (min = 6; max = 4 311) days. Both results demonstrated statistical significance for the global samples (Table 2).

**Table 2:** Average timespan between the RTA date and final PIA date and the medico-legal consolidation date.

Average time (days)	G1	G2 (*n* = 114)	OR	95%CI (Min–Max)
RTA/PIA date				
Global sample (*n* = 114)	676.2 ± 1 021.8	344.1 ± 269.7	1.01	1.00–1.01^a^
Considering 17 cases in G1	2 848.5 ± 1 066.4		1.08	1.01–1.02^a^
Global sample without the 17 cases (*n* = 97)	295.5 ± 239.6		0.99	0.98–1.00
Medico-legal consolidation				
Global sample (*n* = 114)	400.6 ± 766.5	243.1 ± 239.0	1.01	1.00–1.01^a^
Considering 17 cases in G1	1 588.8 ± 1 469.6		1.03	1.01–1.04^a^
Global sample without the 17 cases (*n* = 97)	192.3 ± 194.4		0.99	0.98–1.00

RTA: road traffic accident; PIA: personal injury assessment.

a Significant differences.

Because of the stage of the child’s development when they suffered the RTA, the experts had to delay the last PIA in 17 cases until the next developmental phase (*n =* 10) or growth stabilization (*n =* 7), which corresponded to the end of the pubertal period. In these cases, the time between the RTA and final PIA dates, as well as the medico-legal consolidation date, is described in Table 2, and significant differences are noted. The injuries in these cases included: (i) upper and lower limb fractures (*n =* 13); (ii) traumatic brain injuries (TBI) (*n =* 9); (iii) facial bone fractures (*n =* 4); (iv) severe vertebral fracture/spinal cord injury (*n =* 4); (v) thoracic/abdominal trauma (*n =* 4); (vi) severe tooth lesion (*n =* 1); and (vii) ear trauma with tympanic membrane perforation (*n =* 1). In several cases, the victims had more than one type of injury. Evaluation of the global sample after removing these 17 cases (*n =* 211) showed the average timespan between the RTA date and final PIA date was 321.8 ± 256.9 (min = 28; max = 1 495) days, and the medico-legal consolidation date was 219.7 ± 220.6 (min = 6; max = 1 248) days (Table 2).

We observed several significant differences when considering the victims’ state before the RTA: (i) 39.5% (*n =* 90) presented a pathologic history, including 22.8% in G1 and 56.1% in G2 (OR = 0.2, 95%CI = 0.1–0.4); and (ii) 20.6% (*n =* 47) presented traumatic history, including 6.1% in G1 and 35.1% in G2 (OR = 0.1, 95%CI = 0.1–0.3).

### Temporary outcomes

The temporary outcomes are described in Table 3. The analyses for the *total* and *partialtemporary functional deficit* (without the 17 cases described above) showed the average time in G1 was shorter for both damage parameters. For the *total temporary functional deficit,* the average was 25.4 ± 83.1 days (OR = 0.996, 95%CI = 0.99–1.01), no significant difference was observed. For the *partial temporary functional deficit*, the average was 259.4 ± 456.7 days (OR = 1.001, 95%CI = 1–1.002), and there was a significant difference between the groups. The *quantum doloris* results were similar for G1 and G2, with most victims in both groups assigned grade 3 or 4.

**Table 3:** Temporary outcomes (medico-legal damage parameters).

Items		G1 (*n* = 114)	G2 (*n* = 114)	OR	95%CI (Min–Max)
Temporary functional deficit ($\overline{\mathrm{X}\ }$days)					
Total		14.8 ± 33.6	36.1 ± 112.0	0.996	0.99–1.01
Partial		328.7 ± 609.9	190.0 ± 193.1	1.001	1.00–1.002^a^
Quantum doloris (Grades 1–7)					
$\overline{\mathrm{X}}$		3.7 ± 1.0	3.8 ± 1	0.95	0.7–1.2
*n* (%)	1–2	12.0 (10.5)	11.0 (9.7)	1.10	0.5–2.6
	3–4	81.0 (71.1)	82.0 (71.9)	0.96	0.5–1.7
	5–7	21.0 (18.4)	21.0 (18.4)	1.0	0.5–2.0

a Significant differences.

### Permanent outcomes

Using the *three-dimensional methodology*, the description of the permanent outcomes is presented in Table 4, and the severity degree is presented in Table 5. G1 demonstrated a greater chance of evolving without any of the three sequelae levels assessed (*body*, *functions*, and *situations*), with ORs of 2.8, 3.4, and 3.6, respectively. G1 also showed 50%, 80%, 70%, and 50% increased chances of evolving with minor severity for *body*, *functional* and *situational* outcomes, and *damage coefficient,* respectively. Few *permanent needs* related to the RTA were noted overall, with most individuals in G1 and G2 evolving without them.

The medico-legal permanent damage parameters considered in this study are described in Table 6. PFD was assigned in 60.5% of all cases, with a mean of 6.0 ± 12.1 points (min = 0; max = 80). G1 evolved more without PFD (OR = 2.9), and had a lower average than G2 (OR = 0.96). No correlation was found between pathologic and traumatic history and PFD (*P* = 0.6 and *P* = 0.4, respectively) when controlled for age. In addition, *future damage* was assigned in six cases: G1 (*n =* 3; 2.6%) and G2 (*n =* 3; 2.6%). These cases were related to intra-articular fractures or joint instability of the hip (*n =* 2), knee (*n =* 3), and ankle (*n =* 1).

**Table 4:** Permanent outcomes (three-dimensional methodology).

Items	G1 (*n* = 114)	G2 (*n* = 114)	OR	95%CI (Min–Max)
Body				
Orthopaedical	36 (31.6)	75 (65.8)	0.2	0.1–0.4^a^
Neurological	10 (8.8)	8 (7.0)	1.3	0.5–3.4
Psychiatric	8 (7.0)	8 (7.0)	1.0	0.4–2.8
Others	21 (18.4)	26 (22.8)	0.8	0.4–1.5
Nonexistent	56 (49.1)	29 (25.4)	2.8	1.6–4.9^a^
Functional				
Carriage, displacement, and transfers	18 (15.8)	54 (47.4)	0.2	0.1–0.4^a^
Cognition and affectivity, and communication	25 (21.9)	25 (21.9)	1.0	0.5–2.0
Manipulation and grip	9 (7.9)	31 (27.2)	0.2	0.1–0.5^a^
Ingestion	4 (3.5)	3 (2.6)	1.3	0.3–6.2
Sense	2 (1.8)	3 (2.6)	0.7	0.1–4.0
Sphincter’s control	4 (3.5)	1 (0.9)	5.1	0.5–37.3
Chronic pain	9 (7.9)	12 (10.5)	0.5	0.3–1.8
Nonexistent	66 (57.9)	33 (28.9)	3.4	1.9–5.8^a^
Situational				
Acts of daily living	16 (14.0)	52 (45.6)	0.2	0.1–0.4^a^
Affective, social life, and leisure activities	28 (24.6)	44 (38.6)	0.5	0.3–0.9^a^
Nonexistent	69 (60.5)	34 (29.8)	3.6	2.1–6.3^a^
Permanent needs				
Third‐party assistance (partial or total)	1 (0.9)	8 (7.0)	0.1	0.01–0.95^a^
Regular medical treatments	5 (4.4)	7 (6.1)	0.7	0.2–2.3
Regular medical appointment	12 (10.5)	10 (8.8)	1.2	0.5–3.0
Medication	3 (2.6)	5 (4.4)	0.6	0.1–2.5
Orthoses	2 (1.8)	4 (3.5)	0.5	0.1–2.7
Technical aids	4 (3.5)	5 (4.4)	0.8	0.2–3.0
Prothesis	2 (1.8)	5 (4.4)	0.4	0.1–2.0
Consumables	3 (2.6)	1 (0.9)	3.1	0.3–29.8
Ancillary exams	2 (1.8)	2 (1.8)	1.0	0.1–7.2
Nonexistent	100 (87.7)	93 (81.6)	1.6	0.8–3.4

Note: Body, functional and situational outcomes, and permanent needs categories were not mutually exclusive.

a Significant differences.

## Discussion

We observed several significant differences between children and young- and middle-aged adults. Children’s cases showed better results for the severity of *body*, *functional* and *situational* levels, and permanent damage parameters. These findings are discussed below.

### Evidence for posttraumatic injury outcomes in children

Paediatric traumatology literature includes ample evidence on this topic [1, 30–36]. However, to our knowledge, nothing has been published regarding the medico-legal context. We did not find any differences between G1 and G2 in temporary damage parameters from a global perspective. This was expected, given that we started with samples matched by ISS. However, G1 showed a tendency for shorter recovery times, which was consistent with the literature [2, 34, 37, 38]. This finding contradicted a previous study performed by our team [20], which found that children (*n =* 56) presented a significantly longer* total temporary functional deficit* (days of hospitalization) than adults (*n =* 431) (*P* = 0.03). However, this discrepancy may be explained by the inclusion of a severe case related to a child in a vegetative coma in that study, which deviated from the mean values for temporary outcomes. Although it is known that temporary damage is more frequently lower in children than in adults, this aspect is linked to significant difficulties in performing PIA in this age group; i.e. determining the date of the final PIA and date of the medico-legal consolidation (which may or may not correspond). Given its relevance and particularities, this topic is discussed later.

No difference was found in *quantum doloris* between G1 and G2. Remarkably similar results were noted in both groups, which may be attributed to the use of the ISS-matched samples. Regarding permanent outcomes, although the groups presented similar injury severity in the acute phase (Table 1 and Figure 1), a global evaluation showed G1 evolved better than G2 (Tables 4–6), as noted in the literature [20, 21].

The *three-dimensional* damage assessment revealed the following results. (i) *Body* sequelae were less frequent and had minor severity in G1. These events were nonexistent in 49.1% of the examined cases. Most body damage cases were orthopaedic, similar to results previously noted for general RTA cases [20]; however, these events were 80% less common in G1 than in G2. (ii) *Functional* outcomes were not present in 57.9% of G1 cases. In the other cases, outcomes related to motor function were less common and less severe than those noted in G2, and most other capacities included few cases. (iii) *Situational* outcomes were absent in 60.5% of G1 cases. When present, these outcomes were less common and less severe than those noted in G2. (iv) The *damage coefficient* showed that G1 had a 50% greater chance of evolving better than G2. The standardized difference (Table 5) revealed that the groups were no longer balanced for severity in the permanent damage period. Considering the permanent medico-legal damage parameters, we found that G1 cases had a 190% greater probability of evolving without PFD or with a minor PFD average than G2 cases, which was consistent with the above discussion, thereby reinforcing this evidence.

**Table 5:** Severity of permanent outcomes (three-dimensional methodology).

Severity (0–4)	G1 $\overline{\mathrm{X}}$	G2 $\overline{\mathrm{X}}$	OR	95%CI (Min–Max)	SD
Body sequelae	0.7 ± 0.9	1.3 ± 1.1	0.5	0.4–0.7^a^	−0.60
Functional outcomes	0.1 ± 0.4	0.5 ± 0.8	0.2	0.1–0.5^a^	−0.63
Situational outcomes	0.1 ± 0.4	0.5 ± 0.9	0.3	0.1–0.5^a^	−0.57
Damage coefficient	0.7 ± 0.7	1.0 ± 0.9	0.5	0.4–0.8^a^	−0.37

a Significant differences.

**Table 6:** Permanent medico-legal damage parameters.

Parameter		G1 (*n*=114)	G2 (*n*=114)	OR	95% CI (Min–Max)
Functional deficit					
$\overline{\mathrm{X}}$	(0–100 points)	3.9 ± 10.1	8 .0± 13.5	0.96	0.93–0.99^a^
*n* (%)	0	59 (51.8)	31 (27.2)	2.9	1.7–5.0^a^
	1–9	43 (37.7)	56 (49.1)	0.6	0.4–1.1
	10–19	7 (6.1)	17 (14.9)	0.4	0.1–0.9^a^
	20–39	3 (2.6)	5 (4.4)	0.6	0.1–2.5
	≥40	2 (1.8)	5 (4.4)	0.4	0.1–2.0
Aesthetic damage					
$\overline{\mathrm{X}}$	(Degree 1–7)	1.1 ± 1.3	1.2 ± 1.4	1.1	0.9–1.3
*n* (%)	1	23 (20.2)	25 (21.9)	0.9	0.5–1.7
	2	19 (16.7)	23 (20.2)	0.8	0.4–1.6
	3	8 (7.0)	7 (6.2)	1.2	0.4–3.3
	4	7 (6.1)	8 (7.0)	0.9	0.3–2.5
	5	2 (1.8)	3 (2.6)	0.7	0.1–4.0
	Nonexistent	55 (48.2)	48 (42.1)	1.3	0.8–2.2
Repercussion on sporting and leisure activities					
$\overline{\mathrm{X}}$	(Degree 1–7)	0.4 ± 1.0	0.4 ± 1.3	1.02	0.8–1.3
*n* (%)	1	1 (0.9)	4 (3.5)	0.2	0.03–2.2
	2	7 (6.1)	2 (1.8)	3.7	0.7–18.0
	3	6 (5.2)	2 (1.8)	3.1	0.6–15.8
	4	2 (1.8)	1 (0.9)	2.0	0.2–22.6
	5	1 (0.9)	5 (4.3)	0.2	0.02–1.7
	6	0 (0.0)	1 (0.9)	-	-
	Nonexistent	97 (85.1)	99 (86.8)	0.9	0.4–1.8

a Significant differences.

### Basis for understanding children’s trauma outcomes

In mild or moderate ISS injuries, children evolve with less disability than adults and older adults [1, 3, 6, 30]. Furthermore, younger children recover better after injury than older children [6, 14, 30]. However, disability acquired during childhood is always critical given the potential losses, which depend on the developmental phase when trauma occurs and have long-term implications because of the remaining period of life [1, 2].

Traumatic factors can modify a child’s development and require prolonged vigilance. Trauma can also trigger regression to a previous development stage with loss of acquired capabilities, which may worsen or delay the growth stage. Moreover, trauma can prevent the acquisition of other expected capabilities. For example, in adolescence, which comprised the most common group in this study (59.6%), trauma can cause feelings of inferiority and inappropriate social behaviour. Furthermore, trauma can have wide-ranging impacts on various aspects of life, including school, social activities, and parents’ personal and professional lives. These consequences include absenteeism, changes in educational settings, limited participation in extracurricular activities, and disruptions to parents’ schedules and careers [39–43].

It is also important to note some aspects concerning the type of injuries and respective sequelae in children as follows.

(i) Long bone fractures: these fractures constitute 10%–25% of all paediatric traumatic injuries, and primarily affect the upper limbs [44, 45]. Children’s fractures differ from adults’ fractures because of skeletal immaturity and bone physiology [2, 21]. Fortunately, children have advantages such as remodelling capacity and avoiding long-term deformities [45]. However, some prognostic factors may need to be considered, including [21, 30, 45–47]: (a) the child’s age (the younger the child, the eventually more significant the deformity and dysmetria); (b) energy of the trauma; (c) type and severity of fractures (especially those affecting growth plates, which may disturb the individual’s future growth and development); (d) skin integrity/degree of bone exposure; (e) presence of vascular or nerve branch lesions; (f) quality of fracture reduction (when appropriate); and (g) type of treatment (conservative or surgical). Growth disorders are the most common sequelae resulting from premature growth plate closure or rapid partial growth, leading to shortening or deformity of the affected bone segment.
(ii) Spine fractures and spinal cord injury: children experience more severe spine fractures than adults, as the trauma mechanism required to produce these injuries in children is more forceful [21, 45, 48]. These fractures often affect cartilage growth in the vertebral bodies, leading to scoliosis or kyphosis [21]. In addition, children aged under 8 years are at a higher risk for spinal cord injury without radiological abnormalities [21, 48]. Furthermore, children’s anatomic features, including a proportionally larger and heavier head, mean 75% of cervical spine injuries occur in the upper region [4, 45].
(iii) TBI: brain plasticity means children exhibit a better response and adaptation after TBI than adults [49, 50]. Children are prone to TBI because of their thin skulls and increased vulnerability in RTA when not seated properly in vehicles [5]. Although most TBI in children are minor, those with persistent disabilities can experience significant cognitive and neuropsychological impairments [1, 30, 51]. Psychological or behavioural disorders, as well as cognitive impairments such as executive function disability or memory disorder, are common [6, 52, 53]. In addition, recovery patterns after early TBI in children are unpredictable, meaning it is challenging to identify high-risk cases requiring intensive follow-up and intervention [52, 53].
(iv) Orofacial trauma: dental injuries in children require special consideration because of tooth germs and bone characteristics during childhood. In children aged 1–3 years, trauma to the temporal incisors, deciduous tooth loss (which does not cause any sequelae), dislocations, subluxations, and intrusions are highly prevalent. However, damage to permanent teeth in older children can have critical effects. Dislocations may require reimplantation, which is complicated by root resorption and potential tooth loss [21, 40, 54]. In fractures affecting the maxillary bones, the possible detection of mandibular bone growth should be highlighted. If the fracture occurs before age 12 years, it is necessary to consider the possibility of adjacent tooth germs being affected, with the consequent loss of said tooth pieces. In subluxations, changes in dental germs should be monitored through radiological studies [21, 40, 54].

### Medico-legal difficulties in children’s cases and proposals

Given the complex process whereby trauma affects a person in their growth phase, several difficulties occur in the medico-legal assessment of children’s cases. Some of these difficulties are described below.

(i) Short previous state because of age and limited comparison elements: the evaluation of a person’s current state is always performed by comparing it with their previous state. However, evaluating a child’s current state is challenged by the lack of previous baseline information, especially in infancy and early childhood. Therefore, experts do not have a starting point for assessing specific skills. To overcome this, we consider that it is necessary to: (a) describe the child’s current capacities by comparing them with other children of the same age group without sequelae; (b) seek information to assess development status from parents, family members, and teachers (kindergarten or school, depending on the child’s development status); and (c) explain in the medico-legal report how sequelae may impact future capacities and general competencies in adulthood, considering the current scientific evidence in this subject.
(ii) Establishing the medical causal link: several key aspects must be considered when discussing a medical causal link in children as follows. (a) Children usually present a better evolution of injuries compared with adults, and the anatomical-clinical consistency between the trauma and sequelae (which is fundamental to determining the causal link) may justify particular reasoning in the medico-legal report [24]. (b) Outcomes should be understood and justified considering the child’s growth phase; therefore, medico-legal experts must be aware of the effect of trauma on the growth process and the physiopathology of trauma in children, which can lead to unexpected developments. (c) Medico-legal experts must always consider that some sequelae may not be present at the moment of the expertise but may arise later; in these cases, the final PIA must be postponed until the end of pubertal development (with a regular follow-up until that moment). (d) Determining the timing for establishing a causation link can be challenging. Therefore, experts can initially discuss a preliminary link based on the observed sequelae at a specific moment while emphasizing the need for further evaluations and a reassessment of the causality link at the final PIA.
(iii) Determination of the consolidation date and the time of the final PIA: consolidation is considered when no further evolution of the injuries is expected [24]. This frequently corresponds to the last PIA date or is retrospectively calculated in that final assessment. However, this procedure can be different in children [21], as noted in our study (Table 2) because: (a) in most cases (85.1%; ranging from 7 to 1 134 days—192.3 ± 194.4), the consolidation date aligns with the expected healing time for a specific injury; and (b) in some cases (14.9%; ranging from 47 to 4 311 days—1 588.8 ± 1 469.6) where determining the final sequelae is challenging, the last PIA is postponed to the subsequent developmental phase or final pubertal period. This delay can significantly extend the average closing time of these cases by 827%. Some clinical examples of these cases include: (a) fractures affecting growth plates that require assessment until the final puberty period [21, 55]; (b) spine and spinal cord injuries that require time for motor function recovery until adolescence [4, 56]; (c) certain TBI because neurocognitive recovery can continue even after 10 years [57]; and (d) dental-stomatology injuries that require evaluation of the final dentition growth for prosthesis placement at age 16 or 18 years [21, 40, 54].
(iv) Assessment of permanent damages, including loss of prospective potential and future needs: under civil law in Portugal, compensation processes are typically closed quickly in cases involving children, even in severe cases, because many legal representatives prefer capital compensation and closing the process as soon as possible. However, the option for compensation in rent is legally foreseen and can facilitate clinical follow-up and evaluation of these cases. Furthermore, disabilities in paediatric patients can be challenging to quantify and have more significant temporal implications because of the child’s longer lifespan [2, 21]. Evaluating the long-term impact of trauma on children’s physical, mental, and social development is challenging (if not impossible), as many parameters may not be fully evident at the time of assessment. The responsibility to determine permanent outcomes and needs for a lifetime at a very early stage of a person’s life is challenging to assume. Furthermore, predicting prospective potential can be complex, as children possess untapped potential and many damage parameters that need to be assessed may not yet be present at the final PIA (e.g. those related to sexual and professional aspects). The same occurs with the prediction of *permanent needs*; in our sample, 12.3% of children had *permanent needs*, but this topic is particularly important in severe cases, which is discussed later. To address these challenges, we believe that a reasonable solution involves: (a) delaying the final assessment as much as possible and maintaining long-term surveillance until the individual reaches the end of their growth development period; and (b) anticipating the potential need to reopen the process in future, as outlined in the official Portuguese rules on future damage, and addressing this issue in the medico-legal report [23, 55].
(v) Long-term survival after severe traumatic injuries: estimating the long-term survival of victims with severe sequelae is an aspect that is systematically asked of medico-legal experts. The risk for death in these victims is highest within the first 2 years after the injury and is directly related to their level of disability [58–62]. Factors impacting long-term survival include immobility, severe cognitive, intellectual and communication impairment, compromised self-feeding ability, the need for ventilatory support, and uncontrolled epilepsy. Importantly, modern rehabilitation and good quality care can enhance function and survival and reduce mortality [58–62]. However, a limited number of life/mortality tables are available for use by medico-legal experts, and these tables have not been validated for the Portuguese population. Our proposal on this topic is to: (a) estimate survival time using existing tables (e.g. Traumatic Brain Injury Model Systems funded by the National Institute on Disability and Rehabilitation Research and California Department of Developmental Services [62]) or existing evidence on the matter; (b) promote long-term studies in the Portuguese population, mainly among victims with severe TBI and spinal cord injury; and (c) validate scales that assess the survival time of victims of severe sequelae in the Portuguese population.
(vi) Medico-legal communication with children: marked differences are noted between how children and adults interpret and report on their health. A critical point to consider is how to address children and adolescents, which can be challenging. Age-related differences in cognitive abilities mean some children can interpret and express their health status more clearly than others [2, 36, 45, 63]. Children aged 5 years and older can reliably report pain, complaints, and symptoms, although they may have difficulty quantifying and describing symptom duration [63]. A family member or caregiver should accompany children and younger adolescents during medico-legal visits, especially when the child is unable to fully participate. Although parents often provide valuable insights regarding the impact of the child’s condition on the family, their reports may introduce biassed measurements of their child’s health based on how they have been affected (e.g. because they may occasionally seek increased gains) [19, 43, 50]. Similarly, parents can influence children to exaggerate their difficulties. Therefore, we consider that it is essential to: (a) listen to problems/complaints reported by family members and children separately whenever possible; (b) rigorously and systematically describe these complaints in functional terms and the patient’s real-life situation along with a thorough physical exam, confronting the reported complaints with the results of the physical examination and other eventual ancillary exams to assess the feasibility of these complaints; and (c) use age-appropriate language during assessments to reflect different children’s growth stages.

### High severity cases

As discussed in the Materials and Methods section, one case from our sample was excluded from our analyses because it deviated too much from the median. This case involved a child aged 3 years at the time of the RTA who suffered a very severe TBI with hypoxic-ischaemic encephalopathy and upper limb fracture (ISS = 34). The case evolved to a minimally conscious state. After 4 years of regular medico-legal follow-up examinations, a final PIA was needed. The maximum damage parameters were assigned, and *permanent needs* were considered for regular medical consultations and treatments, medication, consumables, orthoses, technical aids, adaptation of home and transportation and permanent third-party assistance (24/24 hours).

We found two severe cases (1.8%) in our sample of children (PFD ≥ 40 points; Table 6). In these cases, a multidimensional and transdisciplinary assessment of the child and their family should be promoted. The association between the individual’s intrinsic capabilities and the characteristics of their life-space and personal interactions are fundamental in determining the remaining functional abilities and degree of independence and autonomy [64]. Social workers and psychologists are fundamental in considering the reformulation of familial dynamics and the need for earlier psychosocial, educational, and professional support. Living space and transport adaptation experts must also participate in adapting the home, vehicle, and other spaces to promote accessibility and mobility as needed. Rehabilitation professionals are also fundamental to define the various rehabilitation needs, and technical aids are needed to assist the victim’s physical comfort and orientate their family. These evaluations must be made in the real living spaces of the child and will allow medico-legal experts to prepare an objective and valuable assessment report.

## Limitations of this study and further studies

Some limitations of this study were as follows.

(i) The use of a convenience sample with a relatively small size.
(ii) The analyses of temporary outcomes were impaired by the study’s design (ISS matching).
(iii) The delimitation of the study to RTAs and to the Portuguese civil law context.
(iv) The absence of an analysis of the number and duration of medico-legal examinations that the victims underwent as well as concrete difficulties in accessing information on the victim’s previous status.
(v) The absence of a validated medico-legal tool to assess children of each age group. Existing scales have not specifically been validated for paediatric posttraumatic cases.

To better understand these complex cases, further studies are needed as noted below.

(i) A real-world, retrospective, observational, cross-sectional and multicentric study on this subject using a federated data analysis methodology.
(ii) A study focused on a multidimensional and transdisciplinary approach of these cases.
(iii) Studies considering other types of trauma (e.g. sports accidents).
(iv) A validation study of the *Inventory for Handicap Assessment* for children.
(v) A validation study of a long-term survival scale for the Portuguese child population.
(vi) Consideration of guidelines for children’s PIA as described for older adults through the *Consensus Conference on Medico-Legal Assessment of Personal Damage in Older People* [65].

## Conclusions

The present study allowed us to conclude that there are significant differences between children and young- and middle-aged adults, as follows.

(i) Regarding the severity of *body*, *functional* and *situational* levels, children presented better results: (a) no *body*, *functional* and *situational* sequelae were shown in 49.1%, 57.9%, and 60.5% of cases, respectively; and (b) more chances of minor *body*, *functional* and *situational* sequelae were observed in 50%, 80%, and 70% of cases, respectively.
(ii) Children were revealed to have a 50% greater chance of evolving with a minor *damage coefficient*.
(iii) Children presented a 190% greater chance of evolving without PFD and had a low PFD mean (3.9 ± 10.1).
(iv) The average time between the RTA and final PIA date in children’s cases was longer than that in adults’ cases (676.2 ± 1 021.8 days).
(v) The average time for consolidation in children’s cases was longer than that in adults cases (400.6 ± 766.5 days).
(vi) The two previous results were attributable to the need to wait for the next growth phase of the child or to their final pubertal period (*n =* 17), which increases the time for medico-legal PIA conclusion.
(vii) The average time between the RTA and final PIA date and the average time to the consolidation date (without the 17 referred cases) was shorter than in adult cases, but the difference was not significant (295.5 ± 239.6 and 192.3 ± 194.4 days, respectively).

This study underscores the need for more research on this subject and the development of guidelines for children’s PIA based on scientific evidence.

## Data Availability

The results from the dataset are presented in this paper. The full dataset is available from the first author upon reasonable request.
